# AI and clinicians growing together: A cross-sectional survey of clinicians’ attitudes toward AI-CDSS with comparison to 2020 data

**DOI:** 10.1016/j.clinme.2026.100589

**Published:** 2026-04-27

**Authors:** Simona Curiello, Adriane Chapman, Jeremy C. Wyatt

**Affiliations:** a University of Foggia, Department of Social Sciences, Via Alberto da Zara, 11, Foggia, Italy; b University of Southampton, University Rd, Southampton SO17 1BJ, United Kingdom

**Keywords:** Artificial Intelligence, Clinical decision support systems, Clinician attitudes, Cross-sectional survey, Trust in AI, Ethics in healthcare

## Abstract

**Background:**

Clinical Decision Support Systems (CDSS) – software programs that provide patient-specific recommendations to assist in clinical decision-making – have evolved considerably since 2020, with increasing integration of artificial intelligence (AI) and machine-learning features. Modern AI-powered CDSS (AI-CDSS) no longer rely on static algorithms but instead create adaptive, data-driven insights to be used for diagnostics, prognosis and ‍ ‌‍ ‍‌therapy. Exposure in the clinical setting and attention from regulators have increased, yet uncertainty persists about how clinicians view the risks, benefits and integration of such systems within their everyday practice.

**Objectives:**

Our objective in this research was to assess AI‑CDSS perception in 2025 and to explore cross-temporal patterns relative to earlier reports. Specifically, we sought to: (1) examine cross-temporal trends in perceived benefits and harms; (2) describe clinician subgroups by adoption intentions; and (3) examine professional and regulatory concerns following real-world experience with AI.

**Methods:**

A cross-sectional online survey was administered to UK and Italian clinicians (N = 215). The instrument maintained thematic continuity with a 2020 survey while incorporating AI-specific constructs. The questions spanned five themes: perceived advantages, hazards, regulation, clinical utility, and ethical concerns. Cluster analysis (Ward’s method, z-scored items) was used to identify attitudinal clusters. Comparison through time with 2020 data prioritised thematic concordance and relative frequencies.

**Results:**

AI‑CDSS are now more commonly used for diagnostic support (32.6%) compared to primarily administrative purposes in 2020. Greater endorsement was found for AI benefits such as improved diagnostics (63.3%) and medicine management (62.8%). Concerns moved from technological performance to professional issues, such as de-skilling of trainees (59.5%) and automation bias (67%). Regulatory concerns moved from device-focused agencies to evidence synthesis organisations (eg NICE: 31.2%). Hierarchical cluster analysis identified three distinct attitudinal profiles: The Optimists (n = 113), who reported high perceived benefits and low risk; The Balanced Sceptics (n = 83), with moderate scores across dimensions; and The Concerned (n = 19), who reported low perceived benefits and elevated risk perception.

**Conclusion:**

Clinicians tend to display more nuanced, context-specific views of AI‑CDSS following real-world exposure. Clinicians in 2025 reported moderate to high trust in AI-based tools; however, as trust was measured in 2025 only, no direct cross-temporal trust comparison can be made. Persistent concerns regarding ethics, explainability and professional education remain. Specialised regulatory frameworks and training models are needed to optimise safe, effective integration into modern practice.

## Highlights

• Cross-sectional survey of UK and Italian clinicians compared with 2020 data across two independent samples.
• Diagnostic support is now the most reported AI-CDSS use case, up from administrative and drug-dosing tasks in 2020.
• Three distinct attitudinal profiles identified: The Optimists, The Balanced Sceptics and The Concerned.
• Concerns have shifted in emphasis from technical reliability to ethics, explainability and professional autonomy.
• Clinicians favour NICE and NHS England over MHRA for AI-CDSS governance, prioritising evidence over post-market regulation.

## Graphical Abstract

**Figure:**
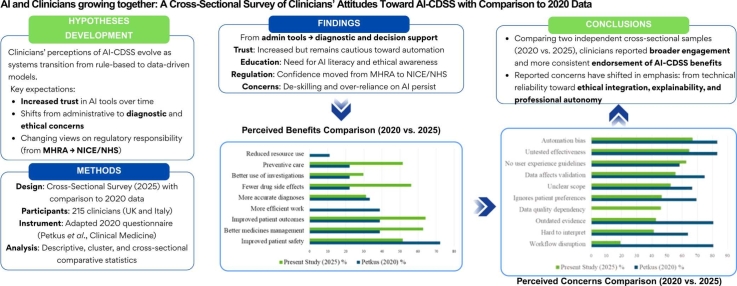

## Introduction

Clinical Decision Support Systems (CDSS) are software programs that provide patient-specific suggestions to assist in clinical decision-making. Their AI-based forms introduce new potential for diagnostic accuracy, medicines optimisation and risk assessment.^1^, ^2^, ^3^ AI-CDSS incorporate a wide range of applications across medical domains: diagnostic systems that support image interpretation and disease detection,^4^ medication optimisation systems that reduce prescribing errors and enhance drug safety,^5^ and predictive tools for the stratification of patient risk and prognosis.^6^ However, alongside their potential, AI-CDSS have created significant challenges in recent years in the areas of explainability, professional autonomy and patient safety.^7^, ^8^, ^9^, ^10^ In 2020, Petkus *et al* conducted a national cross-sectional survey of senior UK physicians through specialty societies to assess attitudes toward AI and CDSS.^11^ The study employed a structured questionnaire using categorical scoring formats and forced-choice selections to evaluate perceived benefits, quality concerns, governance preferences and professional risks. Findings revealed cautious optimism, particularly regarding patient safety and efficiency, alongside concerns about insufficient validation, automation bias, legal risk and regulatory clarity. Their article presented a helpful snapshot of clinician opinion on the threshold of AI interest, aiming at senior physicians and specialty society statements. But since then, experience with AI-CDSS has increased substantially. Use now extends from back-office procedures to front-line decision-taking and has the potential to transform clinical workflows and organisational structures.^12^

By ensuring thematic consistency with the 2020 instrument, this study seeks to illuminate how attitudes towards AI-CDSS have evolved. Compared to the earlier study, which had identified primary quality issues like inadequate testing, outdated evidence and poor advice accuracy, the present research confirms that reliability and the evidence base continue to be major concerns. However, as AI-CDSSs become more ingrained in practice, clinicians are becoming more concerned with usability and alignment with patient care rather than just correctness, as evidenced by the increasing prevalence of interface-related concerns (eg layout variation and indefinite outputs) and patient-oriented issues (eg dominant preferences).

The primary objective of this study was:

1. To assess clinicians’ perceptions of AI-enabled Clinical Decision Support Systems (AI-CDSS) in 2025 and to compare these findings with published results from 2020.
  Secondary objectives were:
2. To examine perceived benefits, risks and regulatory concerns associated with AI-CDSS.
3. To identify distinct clinician attitudinal profiles using hierarchical cluster analysis.
4. To explore shifts in governance preferences and professional concerns following increased real-world exposure to AI-CDSS.

## Methodology

### Study design and purpose

In order to investigate clinicians’ perceptions of risk, expected benefits, and adoption intentions regarding AI-enabled Clinical Decision Support Systems (AI-CDSS), this study used a cross-sectional survey design. A core objective was to enable comparative cross-temporal analysis with the 2020 survey conducted by Petkus *et al*, titled ʻ*What do senior physicians think about AI and CDSS*?’ published in *Clinical Medicine*.^11^ Our study broadens the focus to include value-based perceptions and country-level differences, incorporating a broader European context (UK and Italy), even though both surveys used similar themes and some overlapping constructs.

Importantly, the present study did not follow the same individuals over time. Consequently, rather than being a true longitudinal cohort design, the analysis represents a repeated cross-sectional comparison. Therefore, differences should be interpreted cautiously, as they may reflect contrasts between independent samples rather than true longitudinal change.

**Fig. 1:**
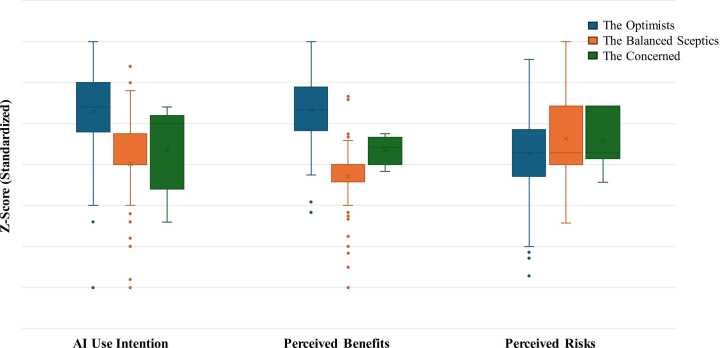
Standardised Z-scores for AI use intention, perceived benefits and perceived risks by clinician cluster.

Source: our elaboration.

Moreover, in order to investigate underlying subgroups of clinicians based on their perceptions of AI-CDSS, we conducted hierarchical cluster analysis with IMB SPSS Statistics (version 27)^1^. Analysis included standardised scores (z-scores) for the constructs: *Perceived Benefits* (12 Likert-scale items); *AI Use Intention* (5 Likert-scale items); *Perceived Risk* (7 Likert-scale items). Variables were standardised to z-scores to weight them equally. We followed Ward’s agglomeration procedure and Squared Euclidean Distance as the similarity measure, respecting best practice in psychological and behavioural research.^13^, ^14^ The number of clusters was determined by considering the dendrogram, agglomeration coefficients and interpretability of solutions. The three-cluster solution was selected based on the following reasons: 1) it showed clear increases in heterogeneity beyond the three-cluster level (seen in the agglomeration schedule); 2) it aligns with the literature suggesting the existence of distinct attitudinal profiles to AI adoption.^15^ The boxplot (Fig. 1) illustrates the distribution of standardised responses across three attitudinal clusters identified through hierarchical cluster analysis.

### Instrument development and mapping to prior study

Our survey was designed to partially replicate and expand upon the instrument utilised by Petkus *et al*.^11^ Although certain response formats were modified to improve granularity (eg Likert scaling replacing categorical or ʻtick up to three’ formats), core constructs and thematic domains were preserved. The comparison therefore emphasises relative frequency patterns, rank ordering and thematic salience rather than direct statistical score equivalence.

Where applicable, to guarantee conceptual comparability, question wordings were aligned to those of the original survey, especially for items on risk, benefit and governance. Although they were not included in the cross-temporal comparison, we also added new items that were in line with value-based healthcare and technology acceptance models (eg UTAUT theoretical framework). To guarantee conceptual comparability with Petkus *et al*,^11^ items were mapped at the construct and domain levels. To increase granularity and psychometric robustness, the current study used Likert-scale responses instead of categorical importance scoring or ʻtick up to three’ formats, which were used in the original survey. Consequently, rather than focusing on direct score equivalency, comparisons highlight relative frequency patterns, rank ordering and thematic salience. A detailed item-level mapping between the 2020 survey^11^ and the 2025 instrument, specifying preserved, adapted and newly introduced items, is provided in Appendix A (Table A3).

The Italian version was produced using forward-backward translation following established questionnaire validation guidelines.^16^ The complete survey instrument, including full item wording and response options, is provided as Supplementary Material 1.

### Data collection and sample

An online survey disseminated through hospital networks, specialty societies and medical associations in the UK and Italy was used to gather data between 14 May and 24 August 2025. Following quality checks, N = 215 valid responses were retained. Similar to the inclusion frame employed in the study by Petkus *et al*,^11^ but not restricted to senior physicians, respondents included clinicians from a variety of specialties.

The survey link was distributed through specialty associations, hospital networks and professional mailing lists in Italy and the UK. A precise response rate could not be determined because distribution took place through institutional mailing lists and third-party networks without access to the entire recipient count. Exclusion criteria included incomplete surveys (defined as >30% missing responses) and submissions failing basic data quality checks (eg patterned or inconsistent responses). As participation was voluntary, self-selection bias cannot be completely ruled out because clinicians with stronger views or greater interest in AI may have been more likely to participate.

Closed-ended answers were exported from Excel and SPSS. A detailed mapping of instrument themes and continuity with the 2020 survey is provided in Appendix A (Tables A1–A2).

Free-text answers from ʻ*other*’ clinical use cases and spontaneous comments were inductively analysed through thematic analysis. Two researchers coded independently and agreed on recurring themes for non-clinical applications (eg patient communication, psychological support). Discrepancies were resolved through discussion.

While the study by Petkus *et al* collapsed thematic outputs into top-level categories, our qualitative analysis was facilitative, used mainly to identify emerging categories poorly addressed by structured items.

The research plan was assessed and approved by the Faculty Ethics Committee (FEC) of the University of Southampton [ERGO No.: 103435]: participation was anonymised and voluntary. No individual or institutional information that could be identified was collected.

Although assuring thematic consistency with previous work published by Petkus *et al*, our analysis contrasted in utilising forced choice and Likert scale responses, which tend to produce greater general frequencies. For this reason, comparisons accentuate rank ordering and relative frequency, rather than score-to-score correspondence. Petkus *et al* only utilised the UK and older physicians, while our sample includes multiple professions and two nations. This might be a less homogeneous but more representative clinical view.

Despite these limitations, cross-temporal analysis is justified due to retention of thematic consistency and item structure, which supports conclusions on cross-temporal contrasts in reported attitudes towards CDSS (rule-based *vs*. AI-enabled) over time.

## Results

### Participants characteristics

A total of 215 clinicians were retained in the final analysis. The sample comprised 57.7% female and 41.4% male respondents. Most participants were aged *≥*50 years (51.1%). The largest professional groups were medical doctors (34.6%) and nurses (39.3%). Clinical experience was fairly evenly distributed, with 30.2% having more than 30 years of experience. Responses were received from both the UK and Italy; however, a country-level breakdown, specialty/subspecialty distribution and work setting are not reported in detail here as they were not collected in a structured format comparable to the 2020 study. These gaps are acknowledged as limitations that constrain direct generalisability and sample comparability.

### Cluster analysis

Hierarchical cluster analysis identified three distinct groups of clinicians based on standardised scores (z-scores) for Perceived Benefits, Perceived Risks and AI Use Intention dimensions (Table 1):

- *Cluster 1*, the most numerous (n = 113), can be typified as ʻ***The Optimists***’. This group showed positive standardised scores across perceived benefits and AI use intention, and negative standardised scores for perceived risks;
- *Cluster 2* (n = 83), termed ʻ***The Balanced Sceptics***’, has features including medium standardised z-scores close to the sample mean across most variables, with slightly lower perceived benefits and AI use intention and slightly higher perceived risks relative to Cluster 1;
- *Cluster 3* (n = 19), the ʻ***The Concerned***’ or the ones referred to as the ʻOpponents’, are not favourable on AI across all dimensioned beliefs. The group showed negative standardised scores across perceived benefits and AI use intention, and positive standardised scores across perceived risks.

**Table 1:** Cluster analysis.

	Variable	The Optimists	The Balanced Sceptics	The Concerned
**Perceived Benefits**	Improve diagnostics	0.68	−0.28	−1.6
	Enhance prognosis	0.67	−0.29	−1.5
	Advance patient management	0.63	−0.32	−1.13
	Accurate care planning	0.74	−0.26	−1.48
	Recommend treatment	0.69	−0.3	−1.56
	Reduce healthcare costs	0.55	−0.18	−1.31
	Improve patient safety	0.64	−0.25	−1.73
	Improve medicines management	0.67	−0.26	−1.53
	Better use of investigations	0.66	−0.27	−1.54
	Preventive care	0.57	−0.3	−1.51
	Reduce drug side effects	0.7	−0.28	−1.14
	Boost outcomes	0.76	−0.31	−1.68
**Perceived Risks**	General risk	−0.5	0.1	1.2
	Uncertainty	−0.4	0.2	1.1
	Potential loss	−0.6	0	1.3
	Unexpected problems	−0.3	0.3	1
	Adverse consequences	−0.5	0.2	1.2
	Patient risk	−0.4	0.1	1.3
	Privacy risk	−0.6	0.1	1.1
**AI Use Intention**	AI Use Intention (mean)	0.92	−0.25	−0.53

One-way ANOVA results were analysed to investigate differences between clusters. Statistically significant differences between clusters were found for all perceived benefit variables and all AI use intention variables (p < .001). For perceived risk variables, Cluster 3 showed significantly higher mean values than Clusters 1 and 2 across general uncertainty, potential loss, unexpected problems, adverse consequences, patient harm and privacy risk (p < .01 to p < .001). Post-hoc Tukey tests revealed significant differences between pairs of clusters for most variables.

These three profiles – distinguished by their markedly different orientations toward AI-CDSS benefits, risks and use intentions – provide the organising framework for the cross-temporal findings reported below and for the engagement strategy implications discussed in the Discussion section.

### Comparative cross-temporal analysis

Diagnostic support was the most frequently reported use (32.6%), followed by interpretation of results (18.6%) and dosage calculation (14.9%). Risk prediction and chronic monitoring were less frequently mentioned.

**Fig. 2:**
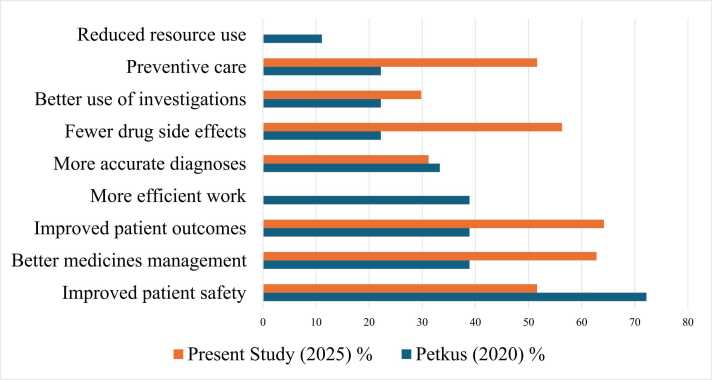
Perceived benefits comparison (2020 vs. 2025).

Source: our elaboration.

In 2025, the majority of respondents agreed or strongly agreed that AI-CDSS improve diagnostic accuracy (63.3%), enhance medicines management (62.8%) and improve overall patient outcomes (63.3%). Improved use of clinical investigation was endorsed by 59.9% of participants. Preventive care received agreement from 51.6% of respondents, while 37.2% selected neutral responses. Reduction of drug side effects was endorsed by 56.3%. A comparison of perceived benefits between 2020 and 2025 is represented in (Fig. 2).

The most frequently reported *Performance & Evidence Quality Concern* was insufficient testing of AI-CDSS effectiveness (64.7%), followed by issues regarding lack of standardisation of interface designs (55.3%), need for high-quality structured patient data (46.0%), potential to ignore patient preferences (46.5%), and the possibility of outdated clinical evidence to support AI-CDSS recommendations (42.8%). Concerns regarding junior staff following AI-CDSS recommendations uncritically were reported by 78.6% of the respondents. Automation bias was also reported by 67.0%. Additional concerns included insufficient expert clinical input into system development (66.0%) and de-skilling of trainees due to over-reliance on automated systems (59.5%).

**Fig. 3:**
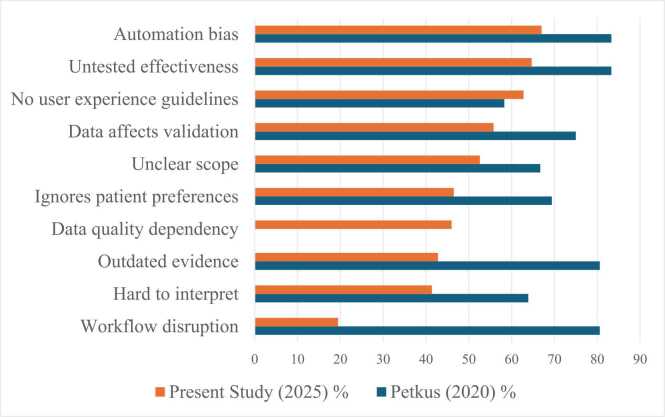
Perceived concerns comparison (2020 vs. 2025).

Source: our elaboration.

Comparative concerns across time are shown in Fig. 3.

Finally, when prompted to identify which bodies ought to set AI-CDSS quality standards, the most frequently selected bodies were NICE (31.2%) and NHS England (28.4%).

## Discussion

The present study offers a cross-temporal perspective on reported clinician attitudes toward AI-supported Clinical Decision Support Systems (AI-CDSS), comparing a 2025 cross-sectional sample with the findings reported by Petkus *et al*.^11^ five years earlier. By preserving thematic consistency across instruments and broadening the geographical and professional scope, we identified noteworthy differences in clinical expectations, reported use patterns, and perceived concerns between the two samples. Beyond cross-temporal comparison, these findings contribute to the broader literature on clinical AI adoption, professional autonomy and digital health governance.

One of the most notable differences concerns the reported positioning of AI-CDSS within clinical workflows. Whereas Petkus *et al*^11^ reported highest use in drug dosing and risk estimation – often downstream and administrative tasks – our 2025 results show a cross-sectional contrast, suggesting greater emphasis on diagnostic and investigative purposes. This difference may reflect greater reported willingness to apply decision support earlier in the diagnostic workflow. It also aligns with technology advancements, with newer tools being designed for real-time, upstream clinical thinking rather than fixed rule-based safety checks.

The contrast between the two samples suggests a difference in reported CDSS application: whereas dosage calculation and prognosis estimation were predominant in 2020, diagnostic and interpretive tasks were more frequently reported in 2025 – a pattern consistent with the broader developmental trajectory from rule-based to AI-driven, data-driven systems. This progression also reflects patterns described in technology adoption frameworks such as the Technology Acceptance Model (TAM) and UTAUT,^17^ where perceived usefulness and workflow integration are central determinants of behavioural intention.^17^ Consequently, the differences in respondents’ reported focus on issues of usability, explainability and professional autonomy are consistent with this general trend.

Compared to 2020, endorsement of AI-CDSS benefits appears more consistent and less fragmented, particularly regarding diagnostics, medicines management and patient outcomes. CDSS problems have remained persistent, but differ in emphasis between the two samples. In 2020, the primary issues were testing, evidence obsolescence and black boxing of CDSS. While these are still issues, 2025 respondents note interface usability, heterogeneity of system design, and non-transparency of validation processes. This emphasis resonates with the expanding literature on explainability and human–AI interaction, which argues that technical performance alone is insufficient without interpretability and workflow coherence. The greater salience of usability as a concern suggests a more pragmatic, practice-oriented perspective among clinicians with increasing exposure to diverse AI tools in routine practice.

The three attitudinal profiles identified through cluster analysis – The Optimists (n = 113), The Balanced Sceptics (n = 83) and The Concerned (n = 19) – point to meaningfully different engagement needs. The Optimists, who reported high perceived benefits and low risk perception, are likely to be early adopters and champions of AI-CDSS; engagement strategies for this group should focus on maintaining critical appraisal skills and avoiding automation bias. The Balanced Sceptics, whose scores clustered around the sample mean, represent the largest group and are likely receptive to targeted evidence-based communication that addresses specific usability and validation concerns before adoption. The Concerned, though the smallest group, expressed consistently negative benefit perceptions and elevated risk concerns; for this profile, engagement requires addressing foundational trust deficits, potentially through direct involvement in AI system co-design and governance. These profiles suggest that a one-size-fits-all approach to AI-CDSS implementation is unlikely to succeed, and that workforce engagement strategies should be tailored accordingly.

Of particular note are the reported differences about impact on junior staff and professional judgement. Recent scholarship on AI and professional autonomy similarly highlights tensions between augmentation and dependency, particularly within training environments. The differences between the two samples in this area – with professional and ethical concerns more salient in 2025 – reflect a maturing of the clinical conversation around AI integration, moving from scepticism about technical performance toward deeper questions about professional identity and education.

A further difference between the two samples concerns preferences for regulatory oversight. Whereas Petkus *et al*^11^ found that trust was largely placed in established institutions such as the MHRA and royal colleges, our 2025 respondents showed a stronger inclination for NICE and NHS England – organisations associated with synthesis of evidence and guideline development, rather than post-market device regulation. This reveals clinician wishes for governance models to be forward-looking, transparent and aligned with clinical guideline development, rather than those entirely centred on safety. This pattern suggests that clinicians may prefer governance models that are forward-looking and aligned with clinical guideline processes, though the cross-sectional design precludes causal interpretation of this difference.

## Conclusions

This study provides cross-temporal contrasts between two independent cross-sectional samples, offering descriptive insight into how reported clinician attitudes toward AI-CDSS may differ between 2020 and 2025. Comparing the two samples, the 2025 data show broader reported application and more consistent endorsement of AI-CDSS benefits – particularly in diagnosis and therapeutic decision-making. Trust was measured directly in 2025 and found to be moderate to high; however, as the 2020 study did not measure trust with the same instrument, no direct cross-temporal inference about trust change can be drawn. Persistent concerns regarding interpretability, professional training, and the changing role of clinical expertise remain prominent in the 2025 sample.

The identification of three distinct attitudinal clusters – The Optimists, The Balanced Sceptics and The Concerned – is perhaps the most actionable finding of this study. These profiles suggest that clinician engagement with AI-CDSS is not homogeneous, and that implementation strategies, training initiatives and governance frameworks will need to be differentiated to be effective. Policymakers and healthcare organisations should consider profiling workforce attitudes as a precondition to AI-CDSS rollout, rather than assuming uniform readiness.

Limitations include differences in survey format between 2020 and 2025, heterogeneous sampling across two countries, voluntary participation, and the inability to calculate a precise response rate – all of which introduce self-selection bias and constrain direct comparability. Findings reflect the views of a self-selected sample and should not be taken as statistically representative. Country-level breakdown, specialty distribution and work setting were not collected in a structured comparable format, further limiting generalisability. Future research should examine routine use barriers, adoption behaviours over time using true longitudinal designs, and how perceptions differ among healthcare systems’ varying levels of digital maturity. Crucially, the professional issues surrounding training and judgement in the age of AI warrant particular attention, given their potential implications for medical education and workforce development.

## CRediT authorship contribution statement

**Simona Curiello:** Writing – original draft, Visualization, Project administration, Methodology, Investigation, Formal analysis, Data curation, Conceptualization. **Adriane Chapman:** Writing – review & editing, Validation, Supervision, Methodology, Funding acquisition, Conceptualization. **Jeremy C. Wyatt:** Writing – review & editing, Validation, Supervision, Resources, Methodology, Conceptualization.

## Ethics approval and consent to participate

This study was reviewed and approved by the Faculty Ethics Committee (FEC) of the University of Southampton, under approval reference [ERGO No.: 03435].

All participants were informed about the study objectives, data protection and voluntary participation before completing the survey. Participation was entirely *voluntary* and *anonymous*, and no identifiable personal or institutional information was collected.

As the study involved clinicians responding to a non-interventional online survey, no patient data, human tissue or clinical procedures were used; therefore, no additional consent beyond voluntary participation was required.

All research procedures were conducted in accordance with the Declaration of Helsinki and the ethical standards of the University of Southampton.

## Funding

This research received no external funding beyond institutional support from the 10.13039/501100000739University of Southampton.

A note of acknowledgement has been included in the manuscript to indicate that the work was partially supported by the 10.13039/100006662NIHR Southampton Biomedical Research Centre.

## Declaration of competing interest

The authors declare the following financial interests/personal relationships which may be considered as potential competing interests: Adriane Chapman reports financial support was provided by NIHR Southampton Biomedical Research Centre. If there are other authors, they declare that they have no known competing financial interests or personal relationships that could have appeared to influence the work reported in this paper.

## Data Availability

The data supporting the findings of this study consist of anonymized survey responses collected from clinicians in the UK and Italy. Due to ethical and confidentiality restrictions imposed by the University of Southampton Faculty Ethics Committee [ERGO No.: 103435], individual-level data cannot be publicly shared. However, aggregated datasets and analysis outputs (eg frequency tables, summary statistics, and cluster analysis code structure) are available from the corresponding author upon reasonable academic request. All analyses were performed using IBM SPSS Statistics (version 27) and standard procedures are described in the Methodology section of the manuscript.

## Footnotes

Appendix A Supplementary data associated with this article can be found in the online version at doi:10.1016/j.clinme.2026.100589.

## Footnotes

1 IBM SPSS Statistics. https://www.ibm.com/products/spss-statistics
