# Supplementary material for: AI and clinicians growing together: A cross-sectional survey of clinicians’ attitudes toward AI-CDSS with comparison to 2020 data
Source: Clin Med (Lond). 2026 Apr 27;26(3):100589. doi: 10.1016/j.clinme.2026.100589 (PMC13195314; doi:10.1016/j.clinme.2026.100589)
Supplement: Supplementary file 1 — Supplementary material [file mmc1.docx]

Clinicians' perceptions of Artificial Intelligence in healthcare

Start of Block: Default Question Block

Q1 This survey is aimed exclusively at medical professionals (surgeons, dentists, pharmacists, psychologists, chemists and physicists, biologists, nurses, nurse practitioners, physiotherapists, radiographers). You have been selected as a possible participant in this study because of your expertise and experience in the medical field. As Artificial Intelligence (AI) is growing rapidly and is likely to become an integral part of healthcare in the future, the perceptions and suggestions of physicians are crucial to guide their governing bodies and researchers in developing and integrating AI-enabled systems into clinical workflows, particularly to assist them.

This short online survey was designed to understand physicians' overall perceptions of AI-based Clinical Decision Support Systems (CDSSs). In particular, this study aims to identify healthcare professionals' expectations of AI in their respective specialties and their willingness to use AI in their routine clinical practice. **Artificial Intelligence (AI) definition** Artificial Intelligence (AI) simply means that a machine (e.g., a computerized system) is able to perform a task that needs physical ability or mental intelligence and previously could only be done by human beings. AI can perform these tasks or make decisions without explicit human instructions.

For example, AI could be a computer application that is good at performing a single task, such as playing chess or poker. Thus, AI could perform a task better than a human.

For the purposes of this research, **AI-based clinical decision support systems** are considered to be automated systems that mimic human cognition and generate results based on past data to assist the user (healthcare professional) in making medical decisions. Examples include automated clinical decision support systems (often integrated with electronic health records), automated image-based diagnosis (mobile apps for skin cancer detection), and machine learning-based predictive models (predicting patient survival, patient readmissions, length of hospital stay, etc.).

Q2 Study Title: **Physicians' perceptions of Artificial Intelligence in healthcare** Researcher(s): Simona Curiello University email: [sc1m25@soton.ac.uk](mailto:sc1m25@soton.ac.uk)

Ethics/ERGO no: 103435

Version and date: V.2; 2025-03-24

**What is the research about?** My name is Simona Curiello and I am a PhD fellow at the University of Southampton in the United Kingdom. I am inviting you to participate in a study regarding healthcare professionals' perceptions, motivations, and challenges in integrating artificial intelligence (AI) medical devices in their clinical practice, with a particular focus on Clinical Decision Support Systems (CDSSs).

This study was approved by the Faculty Research Ethics Committee (FREC) at the University of Southampton (Ethics/ERGO Number: [please insert]).

**What will happen to me if I take part?** This study involves completing an anonymous questionnaire which should take approximately ***15 minutes*** of your time. Participants must complete the survey in one sitting, as their responses will not be saved if they exit before finishing. Please ensure you allocate sufficient time to complete the survey in full before starting. If you are happy to complete this survey, you will need to **tick (check) the box below to show your consent**. As this survey is anonymous, the researcher will not be able to know whether you have participated, or what answers you provided.

**Why have I been asked to participate?** You have been asked to take part because You have been asked to take part because you are a clinician, regardless of your specific professional role, years of experience, healthcare setting (public or private), or clinical specialty. This study aims to gather insights from a diverse range of healthcare professionals to better understand their perceptions of AI adoption in medical decision-making. Participants from various healthcare systems and clinical disciplines are welcome, as the research seeks to explore broad perspectives on AI integration across different medical environments. Your responses will remain **anonymous** and **confidential**, and no identifying information will be collected.

**What information will be collected?** The questions in this survey ask for information in relation to *demographic characteristics* (such as age, gender, years of experience, clinical specialty, and healthcare setting) as well as *perceptions*, *attitudes*, and *concerns* regarding the adoption of AI in clinical decision-making. The survey will explore participants' views on AI-based medical tools, including their *perceived benefits*, *risks*, *challenges*, and *ethical* or *regulatory concerns*. No questions will ask about distressing, sensitive, or personally invasive topics.

**What are the possible benefits of taking part?** If you decide to take part in this study, you will not receive any direct benefits; however, your participation will contribute to knowledge in this area of research.

**Are there any risks involved?** It is expected that taking part in this study will not cause you any psychological discomfort and/or distress, however, should you feel uncomfortable you can leave the survey at any time or contact the following resources for support: *Please list here sources of support which participants could contact, which are appropriate in the context of your research.*

**What will happen to the information collected?** All information collected for this study will be stored securely on a password protected computer and backed up on a secure server. In addition, all data will be pooled and only compiled into data summaries or summary reports. Only the researcher and the supervisor will have access to this information. The information collected will be analysed and written up as part of the researcher’s dissertation and published in a journal. The University of Southampton conducts research to the highest standards of ethics and research integrity. In accordance with our Research Data Management Policy, data will be held for 10 years after the study has finished when it will be securely destroyed.

**What happens if there is a problem?** If you are unhappy about any aspect of this study and would like to make a formal complaint, you can contact the **Head of Research Ethics and Governance**, University of Southampton, on the following contact details: Email: rgoinfo@soton.ac.uk, phone: + 44 2380 595058.

Please note that by making a complaint you might be no longer anonymous.

More information on your rights as a study participant is available via this link: <https://www.southampton.ac.uk/about/governance/participant-information.page>

*Thank you for reading this information sheet and considering taking part in this research.*

Q3 **Please tick (check) this box to indicate that you have read and understood information on this form, are aged 18 or over and agree to take part in this survey.**

- I agree to participate in this study

End of Block: Default Question Block

Start of Block: Demographics

Q4 **Gender**

- Male
- Female
- Non-binary / third gender
- Prefer not to say

Q5 **Age**

- 20 - 29
- 30 - 39
- 40 - 49
- 50 - 59
- 60 or older

Q6 **Ethnicity**

- White
- Black or African American
- American Indian or Alaska Native
- Asian
- Native Hawaiian or Pacific Islander
- Other

Q7 **What is your current professional role?**

- Medical Director
- Clinical Director
- Head of Department
- Consultant (Lead of a Clinical Unit or Specialty)
- Senior Clinical Manager
- Clinical Service Lead
- Junior Doctor (Foundation Year, Core Training, or Specialty Registrar)

Q8 **What is your clinical expertise or specialization?**

- Primary Care / Family Medicine
- Radiology
- Cardiology
- Oncology
- Neurology
- Psychiatry
- Pediatrics
- Surgery (General)
- Orthopedics
- Emergency Medicine
- Pathology
- Dermatology
- Gastroenterology
- Pulmonology
- Endocrinology
- Infectious Diseases
- Rehabilitation Medicine
- Obstetrics and Gynecology (OB/GYN)
- Anesthesiology
- Hematology
- Nephrology
- Rheumatology
- Public Health
- Clinical Research
- Medical Education
- Other (please specify) __________________________________________________

Q9 **What type of insurance coverage applies to your role?**

- Employer-provided Professional Indemnity Insurance (e.g., NHS Clinical Negligence Scheme for Trusts - CNST)
- Self-funded Professional Indemnity Insurance (e.g., through MDU, MPS, or MDDUS)
- Mandatory Medical Malpractice Insurance for Gross Negligence
- Personal Accident Insurance
- Employer-provided Property Insurance (for hospital buildings and facilities)
- Employer-provided Vehicle Insurance (for NHS fleet, ambulances, or work-related travel)

Q10 **How many years of clinical experience do you have?**

- 0 to 5 years
- 6 to 10 years
- 11 to 20 years
- 21 to 30 years
- More than 30 years

Q11 **Have you ever used any AI applications for any reason except for healthcare? (Such as AI embedded in smart devices for any purposes such as financial decision-making)**

- Yes
- No

Q12 **Generally, how familiar are you with an AI application (used for any purposes except for healthcare)?**

- Extremely familiar
- Very familiar
- Moderately familiar
- Somewhat familiar
- Slightly familiar
- Barely familiar
- Not familiar at all

End of Block: Demographics

Start of Block: Block 2

Q13 **Have you ever used any AI clinical applications?**

- No
- Yes

Display this question:

If Have you ever used any AI clinical applications? = Yes

| 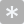 |
| --- |

Q13A **If yes, in which clinical field?**

________________________________________________________________

________________________________________________________________

________________________________________________________________

________________________________________________________________

________________________________________________________________

Display this question:

If Have you ever used any AI clinical applications? = Yes

Q13B **How was your overall experience of using the AI?**

- Extremely challenging
- Very challenging
- Moderately challenging
- Neither easy nor difficult
- Moderately easy
- Very easy
- Extremely easy

Display this question:

If Have you ever used any AI clinical applications? = Yes

Q13C **How did using the AI impact your workload?**

- Significantly increased workload
- Moderately increased workload
- Slightly increased workload
- No change in workload
- Slightly reduced workload
- Moderately reduced workload
- Significantly reduced workload

Display this question:

If Have you ever used any AI clinical applications? = Yes

Q13D **How easy or difficult was it to learn how to use the AI?**

- Extremely difficult
- Very difficult
- Moderately difficult
- Neither easy nor difficult
- Moderately easy
- Very easy
- Extremely easy

Display this question:

If Have you ever used any AI clinical applications? = Yes

Q13E **To what extent did you understand the AI’s output?**

- Did not understand at all
- Understood very little
- Understood somewhat
- Understood moderately
- Understood well
- Understood very well
- Understood completely

Display this question:

If Have you ever used any AI clinical applications? = Yes

Q13F **How meaningful were the AI outputs in your clinical context?**

- Not meaningful at all
- Slightly meaningful
- Somewhat meaningful
- Moderately meaningful
- Quite meaningful
- Very meaningful
- Extremely meaningful

Q14 **To what extent do you think strong statistical knowledge is required to understand the AI?**

- Strongly disagree
- Disagree
- Somewhat disagree
- Neutral
- Somewhat agree
- Agree
- Strongly agree

Q15 **To what extent do you think special training is required to use AI?**

- Strongly disagree
- Disagree
- Somewhat disagree
- Neutral
- Somewhat agree
- Agree
- Strongly agree

End of Block: Block 2

Start of Block: Esmaeilzadeh Constructs

Q16 **Perceived Performance Anxiety**

|  | Strongly disagree | Disagree | Somewhat disagree | Neither agree nor disagree | Somewhat agree | Agree | Strongly agree |
| --- | --- | --- | --- | --- | --- | --- | --- |
| I am concerned that the mechanisms used by AI-based devices may lead to inaccurate predictions |  |  |  |  |  |  |  |
| I am concerned that the mechanisms used by AI-based devices may result in medical errors |  |  |  |  |  |  |  |
| I am concerned that treatments provided by AI devices may be incomplete |  |  |  |  |  |  |  |
| I am concerned that the predictive models of AI-based tools may malfunction |  |  |  |  |  |  |  |
| I am concerned that the medical decisions made by AI devices may be inadequate |  |  |  |  |  |  |  |

| Page Break |  |
| --- | --- |

Q17 **Perceived Social Biases**

|  | Strongly disagree | Disagree | Somewhat disagree | Neither agree nor disagree | Somewhat agree | Agree | Strongly agree |
| --- | --- | --- | --- | --- | --- | --- | --- |
| I am concerned that the mechanisms used by AI-based devices may lead to inaccurate predictions  I am concerned that the AI-based devices may overestimate or underestimate health risks in a certain patient population (e.g., people with insufficient data in AI datasets) |  |  |  |  |  |  |  |
| I am concerned that data used in the AI devices may lead to societal discrimination to a certain patient group (e.g., minority groups) |  |  |  |  |  |  |  |
| I am concerned that AI-based tools used in healthcare may be unfair to a certain group of population (e.g., people with poor access to health care) |  |  |  |  |  |  |  |
| I am concerned that AI devices could lead to morally flawed practices in health care |  |  |  |  |  |  |  |
| Overall, I am concerned that the possibility of biases by AI devices to certain groups of the population is high |  |  |  |  |  |  |  |

| Page Break |  |
| --- | --- |

Q18 **Perceived Privacy Concerns**

|  | Strongly disagree | Disagree | Somewhat disagree | Neither agree nor disagree | Somewhat agree | Agree | Strongly agree |
| --- | --- | --- | --- | --- | --- | --- | --- |
| I think using AI-based applications helps health care entities collect too much personal information from patients |  |  |  |  |  |  |  |
| I think in this case, I am concerned that health care entities use patients' health information for other purposes without their knowledge and authorization |  |  |  |  |  |  |  |
| In this case, I am concerned that patients' health information will be shared with other entities without their explicit consent |  |  |  |  |  |  |  |
| In this case, I am concerned that unauthorized people will have access to patients' health information |  |  |  |  |  |  |  |
| In this case, I am concerned about the privacy of patients' health information during AI-based health practices |  |  |  |  |  |  |  |
| In this case, I am concerned patients' health information would be sold to others without their permission |  |  |  |  |  |  |  |

| Page Break |  |
| --- | --- |

Q19 **Perceived Mistrust in AI mechanisms**

|  | Strongly disagree | Disagree | Somewhat disagree | Neither agree nor disagree | Somewhat agree | Agree | Strongly agree |
| --- | --- | --- | --- | --- | --- | --- | --- |
| I trust in the AI-based clinical tools used for healthcare delivery |  |  |  |  |  |  |  |
| I trust in the AI algorithms used in the healthcare |  |  |  |  |  |  |  |
| I trust in AI 's predictive and diagnostic ability for treatment purposes |  |  |  |  |  |  |  |
| I trust in the accuracy and predictive powers of current AI algorithmic models used in the medical context |  |  |  |  |  |  |  |
| I trust that AI-based tools can adapt to specific and unforeseen medical situations |  |  |  |  |  |  |  |

| Page Break |  |
| --- | --- |

Q20 **Perceived Communication Barriers**

|  | Strongly disagree | Disagree | Somewhat disagree | Neither agree nor disagree | Somewhat agree | Agree | Strongly agree |
| --- | --- | --- | --- | --- | --- | --- | --- |
| I am concerned that AI tools may eliminate the contact between healthcare professionals and patients |  |  |  |  |  |  |  |
| I am concerned that AI tools may reduce conversation between physicians and patients |  |  |  |  |  |  |  |
| I am concerned that AI devices may decrease human- aspects of relations in the medical contexts |  |  |  |  |  |  |  |
| I am concerned that by using AI devices, I may lose face-to-face cues and personal interactions with patients |  |  |  |  |  |  |  |
| I am concerned that by using AI devices, I may be in a more passive position for making medical decisions |  |  |  |  |  |  |  |

| Page Break |  |
| --- | --- |

Q21 **Perceived Unregulated Standards**

|  | Strongly disagree | Disagree | Somewhat disagree | Neither agree nor disagree | Somewhat agree | Agree | Strongly agree |
| --- | --- | --- | --- | --- | --- | --- | --- |
| I am concerned that special policies and guidelines for AI tools are not transparent yet |  |  |  |  |  |  |  |
| I am concerned that the safety and efficacy of AI medical tools are not regulated clearly |  |  |  |  |  |  |  |
| I am concerned that regulatory standards to assess AI algorithmic safety are yet to be formalized |  |  |  |  |  |  |  |
| I am concerned that appropriate regulatory and accreditation system regarding AI-based devices is not in place yet |  |  |  |  |  |  |  |
| I am concerned about the lack of clear guidelines to monitor the performance of AI tools in the medical context |  |  |  |  |  |  |  |

Q22 **Perceived Liability Issues**

|  | Strongly disagree | Disagree | Somewhat disagree | Neither agree nor disagree | Somewhat agree | Agree | Strongly agree |
| --- | --- | --- | --- | --- | --- | --- | --- |
| I am concerned because it is not clear who is responsible when errors result from the use of AI clinical tools |  |  |  |  |  |  |  |
| I am concerned about the liability of using AI-based services for healthcare |  |  |  |  |  |  |  |
| I am concerned because it is not clear who becomes responsible if AI-based tools offer wrong recommendations |  |  |  |  |  |  |  |
| I am concerned because it is unclear where the lines of responsibility begin or end when AI devices guide clinical care |  |  |  |  |  |  |  |
| I am concerned because it is not clear who is responsible if appropriate AI-recommended treatment options are mistakenly dismissed |  |  |  |  |  |  |  |
| Overall, I am concerned that the use of AI clinical tools for clinical purposes increases my liability |  |  |  |  |  |  |  |

| Page Break |  |
| --- | --- |

Q23 **Perceived Risks**

|  | Strongly disagree | Disagree | Somewhat disagree | Neither agree nor disagree | Somewhat agree | Agree | Strongly agree |
| --- | --- | --- | --- | --- | --- | --- | --- |
| The risk of using AI-based tools for medical purposes is |  |  |  |  |  |  |  |
| The degree of uncertainty associated with the use of AI clinical tools is |  |  |  |  |  |  |  |
| The potential loss associated with the use of AI devices is |  |  |  |  |  |  |  |
| The likelihood of unexpected problems with the use of AI devices is |  |  |  |  |  |  |  |
| Overall, the chance of adverse consequences associated with the use of AI-based tools for healthcare purposes is |  |  |  |  |  |  |  |

| Page Break |  |
| --- | --- |

Q24 **Perceived Benefits**

|  | Strongly disagree | Disagree | Somewhat disagree | Neither agree nor disagree | Somewhat agree | Agree | Strongly agree |
| --- | --- | --- | --- | --- | --- | --- | --- |
| I believe AI-based services can improve diagnostics |  |  |  |  |  |  |  |
| I think AI-based devices can enhance prognosis |  |  |  |  |  |  |  |
| I believe AI-based devices can advance patient management systems |  |  |  |  |  |  |  |
| I believe AI-based tools can suggest accurate care planning |  |  |  |  |  |  |  |
| I think AI-based services can recommend reliable treatment options |  |  |  |  |  |  |  |
| I think AI-based tools can reduce healthcare costs |  |  |  |  |  |  |  |
| Overall, I think AI-based devices can boost healthcare outcomes |  |  |  |  |  |  |  |

| Page Break |  |
| --- | --- |

End of Block: Esmaeilzadeh Constructs

Start of Block: UTAUT-2

Q25 **Facilitating Conditions**

|  | Strongly disagree | Disagree | Somewhat disagree | Neither agree nor disagree | Somewhat agree | Agree | Strongly agree |
| --- | --- | --- | --- | --- | --- | --- | --- |
| I think that my centre has the necessary infrastructure to support my use of AI |  |  |  |  |  |  |  |
| I would use AI if I received adequate training |  |  |  |  |  |  |  |
| I would use AI if I received technical assistance when I needed it |  |  |  |  |  |  |  |
| I have resources. (e.g. mobile phone, computer, reporting forms, internet) to use an AI-embedded Clinical Decision Support Systems |  |  |  |  |  |  |  |
| I have the knowledge necessary to use an AI-embedded Clinical Decision Support Systems |  |  |  |  |  |  |  |
| A system like this is not compatible with the way we work |  |  |  |  |  |  |  |
| A specific person (or group) should be available for assistance with difficulties concerning a system like this |  |  |  |  |  |  |  |
| AI-embedded Clinical Decision Support Systems experts are available at any time for assistance with AI application difficulties |  |  |  |  |  |  |  |

| Page Break |  |
| --- | --- |

Q26 **Performance Expectancy**

|  | Strongly disagree | Disagree | Somewhat disagree | Neither agree nor disagree | Somewhat agree | Agree | Strongly agree |
| --- | --- | --- | --- | --- | --- | --- | --- |
| I find AI-embedded Clinical Decision Support Systems more useful in my job |  |  |  |  |  |  |  |
| Using AI-embedded Clinical Decision Support Systems increases my productivity |  |  |  |  |  |  |  |
| Using the equipment enables me to accomplish tasks quickly |  |  |  |  |  |  |  |
| Using the equipment allows me to be more involved |  |  |  |  |  |  |  |
| If I use the equipment, I will increase my chance of improving |  |  |  |  |  |  |  |

| Page Break |  |
| --- | --- |

Q27 **Effort Expectancy**

|  | Strongly disagree | Disagree | Somewhat disagree | Neither agree nor disagree | Somewhat agree | Agree | Strongly agree |
| --- | --- | --- | --- | --- | --- | --- | --- |
| My interaction with AI-embedded Clinical Decision Support Systems is understandable and clear |  |  |  |  |  |  |  |
| Learning on using AI-embedded Clinical Decision Support Systems is easy for me |  |  |  |  |  |  |  |
| It is easy for me to become skillful at using AI-embedded Clinical Decision Support Systems |  |  |  |  |  |  |  |
| In general, I find AI-embedded Clinical Decision Support Systems easy to use |  |  |  |  |  |  |  |
| I expect my interaction with equipment will be understandable |  |  |  |  |  |  |  |
| I expect to find the equipment easy to use |  |  |  |  |  |  |  |
| I expect learning to operate the equipment is or has been easy |  |  |  |  |  |  |  |
| " |  |  |  |  |  |  |  |
| I expect to become skilled at using the AI equipment" |  |  |  |  |  |  |  |

| Page Break |  |
| --- | --- |

Q28 **Social Influence**

|  | Strongly disagree | Disagree | Somewhat disagree | Neither agree nor disagree | Somewhat agree | Agree | Strongly agree |
| --- | --- | --- | --- | --- | --- | --- | --- |
| People who are important to me at work may think that I should use a system like this |  |  |  |  |  |  |  |
| The senior management of this facility will be helpful in the use of such a system |  |  |  |  |  |  |  |
| In general, the facility management will be supportive of the use of a system of this kind |  |  |  |  |  |  |  |
| In general, the district health services management will be supportive of the use of such a system |  |  |  |  |  |  |  |
| My coworkers think that I should use AI-embedded Clinical Decision Support Systems |  |  |  |  |  |  |  |

| Page Break |  |
| --- | --- |

Q29 **Training Adequacy**

|  | Strongly disagree | Disagree | Somewhat disagree | Neither agree nor disagree | Somewhat agree | Agree | Strongly agree |
| --- | --- | --- | --- | --- | --- | --- | --- |
| The training on using AI systems is very helpful in my use of it |  |  |  |  |  |  |  |
| I feel training received is adequate for my efficient use of AI-embedded Clinical Decision Support Systems |  |  |  |  |  |  |  |
| I need another training on AI-embedded Clinical Decision Support Systems to enable me use the system efficiently |  |  |  |  |  |  |  |

| Page Break |  |
| --- | --- |

Q30 **AI Use Intention**

|  | Strongly disagree | Disagree | Somewhat disagree | Neither agree nor disagree | Somewhat agree | Agree | Strongly agree |
| --- | --- | --- | --- | --- | --- | --- | --- |
| I agree to use AI-based tools for clinical purposes |  |  |  |  |  |  |  |
| Using AI-based tools for healthcare purposes is something I would consider |  |  |  |  |  |  |  |
| I would like to use AI-based devices to manage my patients' healthcare |  |  |  |  |  |  |  |
| In the future, I am willing to use AI-based services for diagnostics and treatments |  |  |  |  |  |  |  |
| I am very likely to use recommendations provided by AI-based tools for care planning |  |  |  |  |  |  |  |

End of Block: UTAUT-2
