# Supplementary material for: AI and clinicians growing together: A cross-sectional survey of clinicians’ attitudes toward AI-CDSS with comparison to 2020 data
Source: Clin Med (Lond). 2026 Apr 27;26(3):100589. doi: 10.1016/j.clinme.2026.100589 (PMC13195314; doi:10.1016/j.clinme.2026.100589)
Supplement: Supplementary file 2 — Supplementary material [file mmc2.docx]

**Appendix A.**

**Table A1. Instrument themes and continuity with Petkus et al. (2020)**

| Theme  (Present Study) | Description | Corresponding Theme in Petkus *et al*. (2020) | Type of Adaptation |
| --- | --- | --- | --- |
| Perceived Benefits | Expected advantages of AI-CDSS (diagnostics, medicine management, prevention) | Benefits of CDSS (improved safety, efficiency, outcomes) | Expanded with Likert scale and AI-specific items |
| Perceived Risks | Risks and uncertainties in AI use (accuracy, bias, liability, privacy) | Concerns about CDSS quality and regulation | Reorganized into measurable dimensions |
| Regulatory/  Professional Concerns | Transparency, validation, and professional autonomy issues | Regulation and ethical concerns | Merged and elaborated with newer regulatory actors (e.g., NICE) |
| AI Use Intention | Intention and willingness to use AI in practice | — | New construct (based on UTAUT-2) |
| Performance & Effort Expectancy | Expected usefulness and ease of use | — | New constructs (UTAUT-2 theoretical addition) |
| Training Adequacy & Facilitating Conditions | Availability of support and infrastructure for AI | — | New constructs (implementation context) |

**Table A2. Thematic areas**

| Thematic Area  (2025) | Variables / Codes | Corresponding Construct (2020) |
| --- | --- | --- |
| Clinical Use | Assisting diagnosis; Reporting/interpreting results; Monitoring long-term disease; Calculating dosage; Prognosis | For which clinical tasks do members of your specialty use a CDSS? |
| Perceived Benefits | Improve diagnostics; Enhance prognosis; Advance patient management; Care planning; Medicines management; Preventive care; Reduce side effects; Boost outcomes | Improved safety, efficiency, and preventive care |
| Quality Concerns | Untested effectiveness; Outdated evidence; Layout inconsistency; Data quality dependency; Workflow disruption; Ignores patient preferences | Quality Concerns– reliability of evidence and system transparency |
| Regulatory Concerns | Data affects validation; Unclear scope; No user experience guidelines; No peer-reviewed standards; Testing results not public | Who should set quality standards for CDSS? |
| Professional Risks | Alert fatigue; Automation bias; Uncritical junior use; Reduced training; Lack of expert input | Professional and Ethical Concerns |

**Table A3.** **Item-Level Mapping of Survey Instruments (2020 vs 2025)**

| **Domain** | **Petkus et al. (2020) Item** | **2025 Survey Item(s)** | **Status** | **Comparison Approach** |
| --- | --- | --- | --- | --- |
| **Quality Standards - Setting Authority** | Who should set quality standards for CDSS? (multi-select list incl. MHRA, NICE, RCP, etc.) | Same multi-select list (MHRA, NHS Digital, NICE, NHS England, RCP, Specialty societies, etc.) | **Preserved (minor contextual update)** | Direct frequency comparison (% selecting each body) |
| **Responsibility for Testing** | Whose responsibility should it be to test CDSS? | Same response categories retained | **Preserved** | Direct frequency comparison |
| **Quality Aspects for Evaluation** | “What three aspects…?” (best evidence, ease of use, accuracy, impact, cost-effectiveness, etc.) | Same core items retained; wording clarified | **Preserved with minor wording refinement** | Rank-order comparison of most frequently selected items |
| **Benefits of CDSS (Top 3)** | Tick up to 3 benefits (patient safety, efficiency, diagnostics, etc.) | Likert-scale agreement on equivalent benefit domains (diagnostics, medicines management, outcomes, etc.) | **Adapted (format change)** | Compared using % agreement (“agree/strongly agree”) vs top-ranked frequency |
| **Actions to Realise Benefits** | Tick up to 3 actions (training, testing accuracy, clinician involvement, etc.) | Similar domains included within facilitating conditions & governance items | **Partially preserved; conceptually expanded** | Thematic comparison only |
| **Concerns – Accuracy & Evidence** | Accuracy insufficient; latest evidence; validation testing | Likert-scale items on risk, uncertainty, evidence quality | **Adapted (scaled measurement)** | Compared using relative salience and % endorsement |
| **Concerns – Usability & Workflow** | Layout variation; workflow disruption; interpretability | Expanded Likert items on usability, effort expectancy, clarity | **Expanded** | Thematic comparison |
| **Concerns – Regulation & Standards** | Regulatory rigour; transparency; openness of validation | Perceived Unregulated Standards construct | **Expanded and operationalised** | Construct-level comparison |
| **Concerns – Ethics & Professional Impact** | Bias; black box; legal liability; junior over-reliance; de-skilling | Perceived Liability, Social Bias, Communication Barriers constructs | **Expanded and operationalised** | Construct-level comparison |
| **Use of CDSS in Clinical Tasks** | Tick tasks (diagnosis, prognosis, dosage, etc.) | Same task list retained | **Preserved** | Direct frequency comparison |
| **New Constructs (Not in 2020)** | - | UTAUT-2 (Performance Expectancy, Effort Expectancy, Social Influence, Facilitating Conditions), AI Use Intention | **New (2025 only)** | Not included in cross-temporal comparison |
